# Artificial intelligence applied to laparoscopic cholecystectomy: what is the next step? A narrative review

**DOI:** 10.1007/s13304-024-01892-6

**Published:** 2024-06-05

**Authors:** Agostino Fernicola, Giuseppe Palomba, Marianna Capuano, Giovanni Domenico De Palma, Giovanni Aprea

**Affiliations:** Division of Endoscopic Surgery, Department of Clinical Medicine and Surgery, “Federico II” University of Naples, Via Pansini 5, 80131 Naples, Italy

**Keywords:** Artificial intelligence (AI), Artificial intelligence and laparoscopic cholecystectomy (LC), Deep learning (DL), Cholecystectomy, Bile duct injury (BDI)

## Abstract

Artificial Intelligence (AI) is playing an increasing role in several fields of medicine. AI is also used during laparoscopic cholecystectomy (LC) surgeries. In the literature, there is no review that groups together the various fields of application of AI applied to LC. The aim of this review is to describe the use of AI in these contexts. We performed a narrative literature review by searching PubMed, Web of Science, Scopus and Embase for all studies on AI applied to LC, published from January 01, 2010, to December 30, 2023. Our focus was on randomized controlled trials (RCTs), meta-analysis, systematic reviews, and observational studies, dealing with large cohorts of patients. We then gathered further relevant studies from the reference list of the selected publications. Based on the studies reviewed, it emerges that AI could strongly improve surgical efficiency and accuracy during LC. Future prospects include speeding up, implementing, and improving the automaticity with which AI recognizes, differentiates and classifies the phases of the surgical intervention and the anatomic structures that are safe and those at risk.

## Introduction

Artificial Intelligence (AI) is a discipline that studies models and the development of algorithms so that machines can acquire the ability to self-learn and achieve human-like performances in complex tasks [1]. These algorithms are based on the analysis of large amounts of data, in a limited time span, and the ability to achieve learning, problem-solving, and decision-making in complex scenarios. AI represents, therefore, a tool that with the potential to revolutionize the practice of medicine in many fields, including surgery [2, 3].

Today, medicine has reached a numerous and complex level of data production. Analyzing this complexity of data is not always feasible in a short time and with skills that are difficult to group into a single expert. Therefore, machines with AI capabilities can be a support tool for "observing" the data, learning from the data and supporting the doctors in everyday choices [1]. Currently, AI does not have the goal of replacing the doctor [4].

Initially, the AI was trained to recognize and classify radiologic images, then surgical actions such as sutures and knots [5]. Currently, AI is applied intraoperatively: it recognizes the phases of a surgical procedure and the anatomic regions of interest (ROIs). In this context, AI has found application during laparoscopic cholecystectomy (LC) [6].

Cholecystectomy is the most common operation performed worldwide by general surgeons with between 750,000 and 1,000,000 performed only in the U.S. annually [7]. LC was introduced into clinical practice approximately 30 years ago. It quickly became the gold standard operation for patients with symptomatic gallstones [8].

Despite the significant advantages, iatrogenic injuries can occur during LC [9]. Iatrogenic complications during LC require improved preventive strategies [10]. The difficulties of LC are linked to the recognition of the anatomic structures in case of inexperience of the surgeon or cholecystitis and to the recognition of the operating phase [11, 12]. One of the main complications is bile duct injury (BDI), caused by failure to recognize it by the surgeon [13, 14].

Historically, surgery has been one of the branches most inclined to innovation and adoption of new technologies able to offer advantages in terms of performance [13]. Despite its potential, the development of medical AI systems represents an untapped opportunity, especially in surgery [15, 16].

Some typologies of AI as Machine Learning (ML) and Deep Learning (DL) have been evaluated to overcome the surgical difficulties listed first. In the literature, several articles on this topic are present. To our knowledge, this is the first review that analyzes the application of AI during LC. Our review stems from the observation of the great importance that AI is assuming in recent years. The aim of this review is to evaluate the use of AI in recognizing the operative phases of LC, in identifying specific anatomic regions (at risk and not at risk) during the operation and its application during cholecystitis. Finally, we analyze the future prospects of this technology.

## Methods

We performed a search on PubMed, Web of Science, Scopus and EMBASE using the following keywords: “artificial intelligence and cholecystectomy” AND “artificial intelligence and laparoscopic cholecystectomy” OR “application of artificial intelligence during a cholecystectomy” OR “AI and gallbladder anatomy” OR “AI and future perspectives in cholecystectomy” OR “Future of AI in automated laparoscopic cholecystectomy” OR “AI and surgery of cholecystectomy”. Articles in the literature on AI applied to laparoscopic cholecystectomy from January, 2010 to December, 2023 were analyzed (Table 1). Only English studies were included. Incomplete and non-peer reviewed in preprint articles were excluded. We focused our attention on randomized controlled trials (RCTs), meta-analysis, systematic reviews and observational studies on cohorts of patients. The articles included were either prospective or retrospective, monocentric or multicenter studies, and with a variable number of patients (hundreds to thousands) (Tables 2, 3).

**Table 1:**
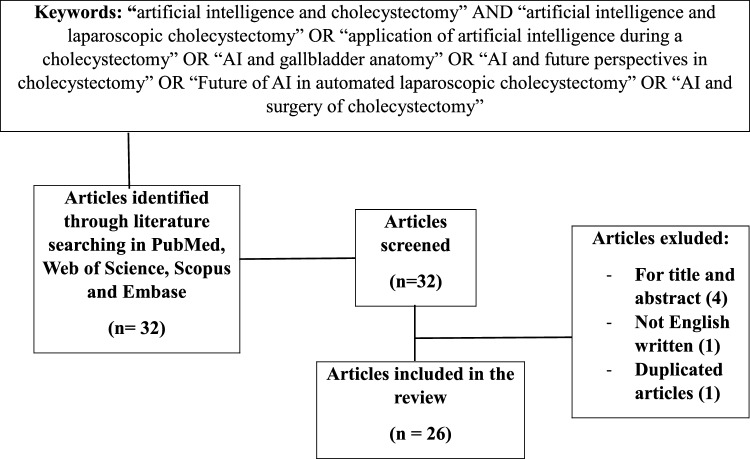
Research flowchart

**Table 2:** A summary of the articles describing the role of AI applied to LC

Authors	Year of publication	Design	Type of study	LC videos numbers
Mascagni et al. [37]	2020	Prospective	Single center	100
Mascagni et al. [38]	2022	Prospective	Single center	201
Madani et al. [39]	2022	Prospective	Multicenter	290
Laplante et al. [40]	2023	Prospective	Multicenter	25
Khalid et al. [6]	2023	Prospective	Single center	22
Endo et al. [41]	2023	Prospective	Single center	20
Nakanuma et al. [42]	2023	Prospective	Single center	10
Fujinaga et al. [43]	2023	Prospective	Single center	92
Kawamura et al. [36]	2023	Prospective	Single center	72
Golany et al.[44]	2022	Prospective	Multicenter	371
Cheng et al. [48]	2022	Retrospective	Multicenter	90

**Table 3:** A summary of the articles describing the role of AI applied to LC

Authors	Year of publication	Dataset and characteristics of the methods	Algorithm and AI
Mascagni et al. [37]	2020	78 endoscopic videos of consecutive LC procedures performed at the *Digestive Surgery Department of the Nouvel Hospital Civil*	The inter-observer agreement was calculated with Cohen’s kappa; doublet view method and the binary scheme
Mascagni et al. [38]	2022	2854 images from 201 LC videos were annotated and 402 images were segmented	The inter-observer agreement was calculated with Cohen’s kappa
Madani et al. [39]	2022	308 anonymized LC videos from 37 countries (including all continents), 153 surgeons and 136 different institutions AI predictions were evaluated using tenfold cross-validation against annotations by expert surgeons. A tenfold cross-validation technique evaluated the performance of GoNoGoNet and CholeNet	*ffmpeg 4.1 software* extracted frames from videos; *Deep Convolutional Neural Network* (CNN; ResNet50); *Pyramid Scene Parsing Network* (PSPNet) was used for pixel-wise semantic segmentation *GoNoGoNet* AI and *CholeNet*
Laplante et al. [40]	2023	308 anonymized videos from 37 countries, 153 surgeons and 136 different institutions High-volume expert surgeons delineated the boundaries of the Go and No-Go zones (semantic segmentation)	*ffmpeg 4.1* software extracted frames from videos; *Think Like A Surgeon software* was used by expert surgeons to delineate the boundaries of the Go and No-Go zones *Visual Concordance Test (VCT)* calculated the expert consensus of annotated pixels for Go and No-Go zones *GoNoGoNet* AI
Khalid et al. [6]	2023	The database was composed of 308 anonymized LC videos originating from 37 countries across 153 surgeons and 136 different institutions. Two groups: BDI group and Control group. 11 LC videos of BDI group was annotated by GoNoGoNet and compared to another 11 LC videos with cholecystitis of control group	*Convolutional Deep Neural Network* (DNN); *U-Net architecture* (the optimized version of the *GoNoGoNet algorithm*);
Endo et al. [41]	2023	230 videos of LC conducted from 2019 to 2021 in a single center; 95 cases that remained on video with mild inflammation to be noted; cases with severe inflammation and abnormal biliary anatomy were excluded 1754 images from Calot triangle dissection scenes, 1610 images of LM-EHBD, 1503 images of LM-EHBD, 1623 images of LM-S4, and 1505 images of LM-RS Test datasets: 19 cases, comprising 190 images of LM-EHBD, 186 images of LM-EHBD, 192 images of LM-S4, and 190 images of LM-RS Questionnaire survey was given to beginners and experts surgeons	*YOLOv3*
Nakanuma et al. [42]	2023	10 cases of LC performed in a single center to evaluate the feasibility of LC using an intraoperative AI-assisted landmark recognition The landmark detection system was connected to the endoscopic integrated operating room (EndoALPHA; Olympus Corp.) The method for quantitatively evaluating the accuracy of landmark detection by AI was the DICE coefficient. The DICE coefficient is the reference value for detecting the accuracy of AI and shows the degree of agreement between expert surgeons for the annotations of the anatomic regions of interest The *Kruskal–Wallis test* was used to evaluate *the DICE coefficient*	*YOLOv3* connected with the endoscopic integrated operating room (*EndoALPHA; Olympus Corp.)*
Fujinaga et al. [43]	2023	20 cases of LC performed in a single center External evaluation committee (EEC) evaluated the adequacy of the detection times of the landmarks detected using AI 4-point rubric questionnaire was used to evaluate the accuracy of landmark detection and the contribution of cross-AI in preventing BDI	*Cross-AI system* for landmark detection and surgical phase recognition: an endoscope (*OLYMPUS LTF-S190-10*; Olympus Corp., Tokyo, Japan), a video processor (*VISERA ELITE II*; Olympus Corp.), and a desktop computer that had two graphics processing units (*Quadro RTX 6000* and *Quadro RTX 4000*; NVIDIA Corp., Santa Clara, CA, USA)
Kawamura et al. [36]	2023	72 LC videos, and 23,793 images were used for training data, performed in a single center	*EfficientNet-B5:* a CNN to classify images and perform prediction; *Sharpness-Aware Minimization*: a SAM an optimizer that perfects the learning parameters of *EfficientNet-B5* to smooth the information derived from the diversified images for a label
Golany et al.[44]	2022	371 LC videos from 4 hospitals and data set Cholec80: 294 videos were used to train the AI model, 77 videos were used to test the AI model. Each video was divided into 10 steps by 2 expert surgeons Experienced surgeons noted the phases, adverse events and CSV of LCs The inter-rater agreement score was used to confirm the quality of the annotations	*MS-TCN—Multi-Stage Temporal Convolution Network*. is a set of temporal convolution layers that capture temporal connections. The final layer of the MS-TCN gives the prediction of the surgical stage for each frame of the LC video *Resnet50* independently classifies and predicts the surgical phase. For each frame (input), *Resnet50* produces a numerical vector of visual features. The set of input vectors are combined to form a sequence of feature vectors representing the entire LC video and fed into the MS-TCN model
Cheng et al. [48]	2022	Dataset: 163 LC videos collected from four medical centers. 90 videos were labeled by expert surgeons. 63 LC videos were used to test the AI model The mean concordance correlation coefficient was used to compare the reliability between the two surgeons	*Super Video Converter software* (version 1.0); The *Anvil Video Annotation Research Tool software* was used to annotate the videos; *FFmpeg software* was used to extract frames from videos; *Convolutional neural network* (CNN) captured the spatial characteristics; *Long Short-Term Memory* (LSTM) analyzed the temporal information *Anaconda* (Anaconda, Inc, Austin, TX) in Python 3.6.5. *NVIDIA* (NVIDIA, Santa Clara, CA) *Tesla V100* graphics processing unit (GPU) trained the visual and temporal models

**Table 4:** Terminology of Artificial Intelligence

*Machine Learning* Subfield of AI that focuses on the development of algorithms and statistical models that allow computers to learn and make decisions or predictions based on data	
*Deep Learning* Subfield of ML that focuses on different layers of neural networks in order to recognize complex patterns, learn from data, and make decisions without having been explicitly programmed. The term "deep" refers to the depth of neural networks that allows them to extract a hierarchy of features from raw input data	
*Natural language processing* Type of AI that have to teach computers how to analyze human language	
*Computer vision* Subfield of AI that allows machines to derive and interpret information from visual data, images and videos	
*Expert system* Computer program that uses AI to simulate the judgment and behavior of a human being or a group of human beings with specific expertise in a particular field of interest	
*Neural networks* Computer system that are designed to mimic the functions of the human brain	
*Decision trees* Graphical representation of decision-making process	
*Support vector machines* ML algorithm used for classification and regression analysis	
*Data mining* The revelation of patterns and insights in large datasets	

Our main outcome is to evaluate the current various studies regarding the association between AI and LC. Our secondary outcome is to evaluate any future prospects for the application of AI in the same field of interest.

From a total of 32 articles analyzed, 26 items were selected and 6 excluded, as shown in Table 1.

## Terminology of AI

AI has various fields of application and branches of interest (Table 4; Graphic 1–2) [17, 18]. The most developed branch is ML (Graphic 2) [19]. ML uses algorithms that derive answers from a large amount of data and statistical analyses [20]. ML can be supervised or unsupervised. Supervised ML uses a training dataset to teach models to produce the desired output. An algorithm measures the accuracy of the model and corrects it until the desired result: in this way, the AI model improves and learns as time goes by. Examples of ML are neural networks (Graphic 3), linear regression, logistic regression and random forest. Classifiers use algorithms to precisely assign test data into specific categories, while regression uses an algorithm to understand the relationship between dependent and independent variables and are useful for predicting specific numerical values. A practical application of ML is the recognition, classification and categorization of images and videos [18, 21].
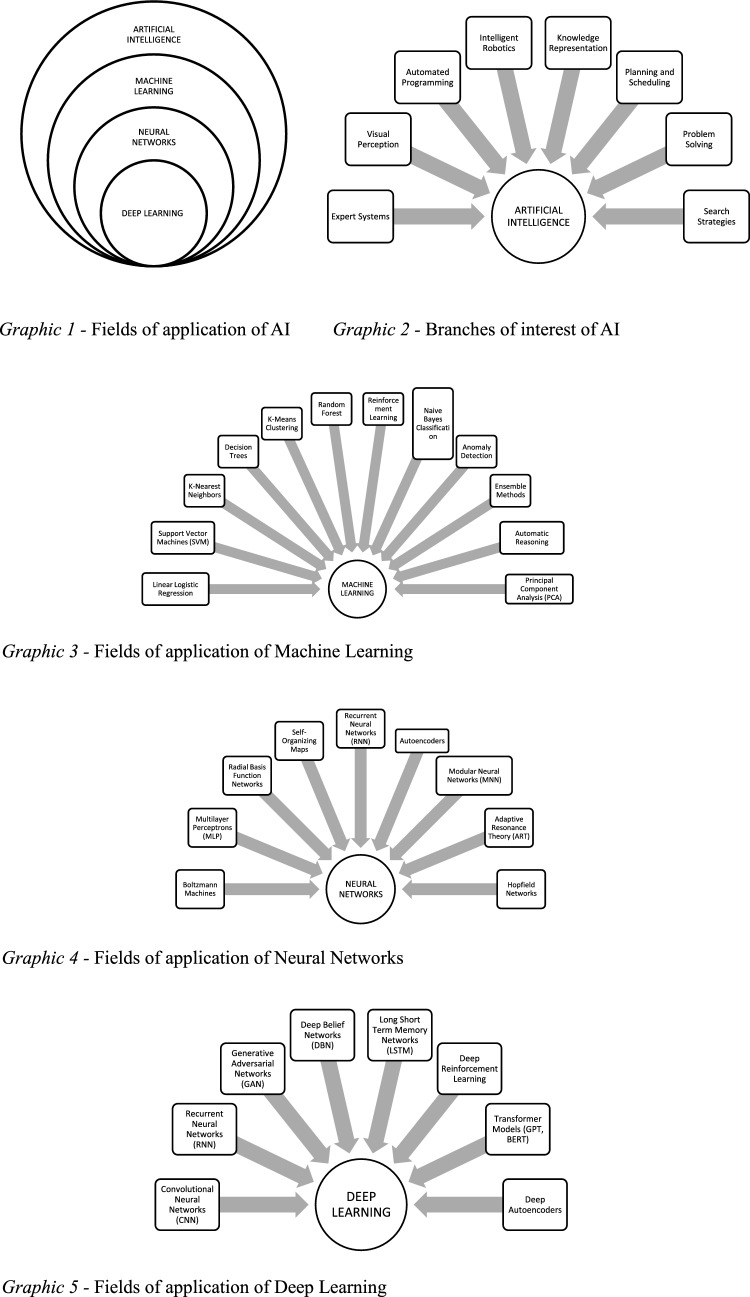


Unsupervised ML does not use predetermined annotations, so it analyzes and clusters previously unlabeled datasets [22]. The main tasks of unsupervised ML are clustering, association and dimensionality reduction. Clustering is a data mining technique that groups unlabeled data based on their similarities or differences. This technique is useful for compressing images and videos. Association uses different rules to find relationships between variables in a given dataset. Dimensionality reduction is used when the number of dimensions or features in a given dataset is too high. It reduces the number of data inputs to a manageable size while preserving data integrity. This is often used during the processing phase of some preliminary data [17]. The fields of application of the Neuronal Networks and DL are shown in Graphs 4 and 5.

## Results

The results of our review have been organized into paragraphs, divided into fields of application of AI during LC. The topics concern the phases of the surgical intervention and the anatomic structures that are safe and those at risk.

### AI and the critical view of safety

Iatrogenic BDI is one of the intraoperative complications of LC [23, 24]. In 1995, Strasberg et al. indicated a surgical strategy to obtain a better intraoperative view of the gallbladder and reduce iatrogenic lesions during cholecystectomy [25]. This surgical approach is called Critical View of Safety (CVS) [26]. It is based on three principles: identification of Calot's triangle and the hepatoduodenal ligament, mobilization of the gallbladder fundus, identification, isolation and section of the cystic duct and cystic artery [27, 28]. The European Association of Endoscopic Surgery (EAES) recommends CVS as the best approach to cholecystectomy [29].

Despite the adoption of intraoperative CVS research, BDI rates remain higher than open cholecystectomy (0.1–0.2%), although decreasing over the years [30]. Some authors, such as Nijssen et al., have hypothesized incorrect awareness and standardized identification of CVS as the cause of this [31]. Nijssen's team analyzed videos of LC performed consecutively from 2009 to 2011 (1108 patients) in their hospital [31]. According to the surgeons' operating notes, CVS was detected in 80% of cases. However, according to the reviewers of these LCs, CVS was achieved in only 10.8% of cases and it was not achieved in any of the patients with BDI. The author concluded his article by highlighting the importance of video analysis of LCs to improve surgical technique and therefore reduce BDI. Rawlings et al. found similar findings in their study: according to the surgeons, CSV was detected in 100% of cases in 54 LCs analyzed, but from the reviewers’ analysis of videos of these LCs, CVS was identified in only 64% of cases [32].

These observations prompted Sanford and Strasberg to develop a method for the correct evaluation of CSV identified by surgeons from both photographic and video images of LCs [33]. The method consisted of a scale with 6 observation points, medial and lateral to the hepatocystic triangle. CVS was correctly identified in 96.5% of videos and 70% of LC images. Budding et al. assessed CVS through still images in only 27% of 63 LC procedures analyzed and reporting weak to minimal inter-rater agreement [34]. The result of these studies is the need to generate methods for the objective evaluation of LC frames or videos to improve both the surgical technique and the identification of CVS [35].

AI models were then created to try to achieve this [36]. Mascagni et al. performed a single-center study on this topic, analyzing the 60 s preceding clipping and cystic structures sectioning of 78 LC videos performed consecutively between March and June 2016 and attempting to document the achievement of CSV [37]. Video reviewers scored medial and lateral visualization of the hepatocystic triangle for each of the three CSV criteria. The authors' study is among the first to use LC videos instead of photographic images for CVS reporting, demonstrating a superiority in the consistency of CVS evaluation from videos compared to that from photographic images. According to the authors, analyzing videos of LCs before sectioning cystic structures could be a starting point for training AI models.

Mascagni et al. developed an AI model to reduce the subjectivity of CSV recognition [38]. According to the authors, the use of a DL model could automate CSV recognition. The ultimate goal was therefore to reduce the rate of iatrogenic injuries during LC. From 201 LC videos, 2854 images were extrapolated and used to train a deep neural network. The AI had to recognize the hepatocystic anatomy and classify the type of anatomic structure. The goal was to be able to predict the achievement of the CSV criteria. The AI was then trained with both a segmentation and classification model. This AI achieved a CSV criteria recognition accuracy level of 71.9% and a precision of 71.4%.

Also Madani et al. trained an AI model to recognize anatomic structures of interest and safe and unsafe areas to perform LC [39]. The authors used 290 videos from American and European hospitals and from each of them they extrapolated 10 frames previously clipping and sectioning the cystic structures. Three expert surgeons, for each frame, noted the possible presence of a specific anatomic structure (liver, gallbladder, hepatocystic triangle), a safe dissection area and an unsafe dissection area. A fourth hepatobiliary surgeon annotator evaluated the annotations applied on the pixels of the frames examined by the three surgeons previously. The safe dissection area (called *Go*) was located within the hepatocystic triangle near the inferior gallbladder border. The unsafe dissection area (called *No-Go*) was the area located most deeply in the hepatocystic triangle, together with the hepatoduodenal ligament and the hepatic hilum, and most likely to injure the cystic duct. The computer vision model obtained was that of semantic segmentation. Classic regions of interest (ROIs) are rectangles and include anatomic ROI. ROIs may have a limited role in the surgical field where structures have more complex geometric configurations and blend into their background. Semantic segmentation allows to delineate an anatomic structure along its exact boundaries pixel by pixel as an overlay on the original video. In fact, the authors superimposed the first model with Go areas on a second model with No-Go areas: these models were together named *GoNoGoNet*. A third model, called *ChiletNet,* simultaneously mapped the location of anatomic structures in each frame. In each of them, each pixel was classified as hepatocystic triangle, liver, gallbladder, or none of the above. To evaluate the performance of the models, the agreement between the expert surgeons' annotations and the AI's predictions of an anatomic structure was measured as semantic classification. For *GoNoGoNet*, the visual results were expressed either as a heat map (red region: highest probability of being in the Go region; blue region: lowest probability) or as a binary format (pixels in the Go region are highlighted and every pixel not in the Go region is not highlighted). Accuracy for pixel identification was greater than 90% for all structures.

The *GoNoGoNet* model was subsequently tested by another group of authors. Laplante et al. fed 47 frames of 25 LC videos to the aforementioned AI model [40]. The annotations applied by six expert surgeons were compared with those of the AI. The authors concluded the study by stating that the AI predictions had high specificity/PPV for Go zones and high sensitivity/NPV for No-Go zones and that the model's prediction was better for No-Go zones than for Go.

The *GoNoGoNet* model was also tested by Khalid et al. [6]. The authors tested the AI on 11 videos in which BDI was present. Another group with 11 videos without BDI was used as a control group. *GoNoGoNet* detected 33.6% more total tissue interactions in the No-Go zones in the BDI group compared to the control group. This meant that the BDI videos showed more dissections occurring outside the safe regions suggested by the AI.

For the first time, Endo et al. have developed an AI model capable of identifying the anatomic structures of interest, during LC: the extrahepatic bile duct (EHBD), the cystic duct (CD), the lower border of the liver S4 (S4) and the Rouviere sulcus (RS) [41]. The authors used images derived from 95 LC videos to train an AI DL model, annotating anatomic structures of interest. The authors excluded inflamed anatomic structures because they were difficult to annotate. An LC video of the 20 s preceding the detection of Calot's triangle was viewed by 4 experienced surgeons and 4 novice surgeons (with fewer than 50 LCs performed as first operator). The eight surgeons noted the anatomic landmarks mentioned above. Subsequently, the surgeons were shown the LC video with the annotations performed by the AI. In 26.9%, surgeons subsequently reformulated their notes, and 70% of these changes were considered safer changes, particularly for EHBD and CD. Subsequently, a questionnaire was submitted to the eight surgeons. In the questionnaire, it asked whether there was any change in each surgeon's perspective after watching the AI-driven video. Half of the interviewees considered the support given by AI in the safe recognition of anatomic structures to be significant.

Nakanuma et al. performed a similar experiment in their hospital center on ten cases of intraoperative LC, using an AI pre-trained to recognize anatomic structures [42]. Subsequently, a group of surgeons evaluated the accuracy of recognition of anatomic structures by the AI (CD, CBD, RS and S4). The structures of interest were delimited by ROIs in the shape of small tiles, of different color based on the anatomic structure. The small tiled automatically reposition and resized to form anatomic landmarks. In this case, the AI model (called *YOLOv3*) was directly connected to the integrated endoscopic operating room (*EndoALPHA*; Olympus Corp.). *YOLOVv3* was installed on a monitor for landmark detection. In the operating room, there was also the main monitor that displays the endoscopic images. Then, the surgeons performed the 10 LCs while observing both monitors. The delay between the surgical video and the video showing the AI tracking information was 0.09 s. For the authors, the use of AI was considered useful for the recognition of anatomic structures of interest. Later, the same group of authors used two AIs simultaneously to identify anatomic landmarks during different phases of LC [43]. One AI had to recognize the surgical time during LC and the other AI had to recognize the anatomic structures of interest. Again, the goal was to evaluate an AI model's ability to prevent BDI. The authors used 1826 images from 92 LCs to train the AI to recognize CD, CBD, RS and S4. The annotations of the different surgical phases and anatomic structures were made by two surgeons with at least 10 years of experience. The two surgeons performed approximately 10 thousand notes. According to the authors, the dual AI system recognized anatomic structures in 92% of LC steps.

Recently, Kawamura et al. [36] have developed an AI capable of reporting CSV intraoperatively during LC. The authors used 72 videos, excluding cases of severe cholecystitis, as the identification of anatomic structures was more difficult and excluding LCs that ended in open cholecystectomies. From the videos, 71,379 images were obtained for annotation. Also in this study, two doctors (surgeon and gastroenterologist) made the annotations to train the AI to identify CSV. The AI was trained to automatically predict the anatomic ROI in which to find CSV. 20 of the 72 LC videos were used for data evaluation. The mean values of overall precision and accuracy were 0.971 and 0.834, respectively.

### AI and recognition of cholecystectomy times

In addition to the application of AI in the recognition of anatomic structures during LC, some authors have trained AI in the recognition of surgical phases in LC videos. In particular, Golany et al. have developed an AI system (called *Resnet50*) capable of recognizing surgical phases during complex LC, such as in case of adverse events and complications [44]. The authors used 371 videos, of which 80% to train the AI and the remaining 20% to evaluate the AI. A group experienced surgeons (experience level more than 20 years) noted 8 steps for each LC video: trocar insertion, preparation, dissection of Calot's triangle, cropping and cutting, gallbladder dissection, gallbladder packing, cleansing and hemostasis, and gallbladder extraction. Beyond these eight phases, the authors defined the phase in which the instruments did not perform any surgical movement as "inactive" and the phase in which the camera was outside the patient's body as "out of body". LC videos considered complex were those with major bleeding, gallbladder perforation, major bile leak. Finally, a score was assigned for the difficulty of each surgical procedure, based on the *Parkland Grading Scale* [45, 46]. Finally, a final score was assigned for achieving the CSV. After the training, the AI had to be able to classify the surgical phase of the LC in which the given input (video frame) administered was located. To temporally classify the correct sequence of surgical stages, the authors integrated a variant of AI *Cholec80*, the so-called *Multi-Stage Temporal Convolution Network* (MS-TCN) [47]. *Resnet50* achieved a classification accuracy of 78%. *MS-TCN* achieved a classification accuracy of 89%. The best results were obtained for the Calot's triangle dissection, clipping and cutting, and gallbladder dissection phases. The worst results were obtained for the preparation phase because it was confused with the dissection phase of Calot's triangle. Furthermore, as the complexity of the LC video increased, the accuracy of the model decreased, from 92% for lower complexity levels to 81% for higher complexity levels. According to the authors, adverse events during cholecystectomy influenced the accuracy of AI in recognizing surgical phases. In videos without adverse events, accuracy averaged 90%. In videos with cholecystitis, accuracy was 89%, in those with gallbladder perforation it was 87%, in those with major bile leak it was 77%. LC videos were extracted from 5 different hospitals to evaluate any difference in AI accuracy in identifying surgical phases during cholecystectomy. The AI was trained on videos from four hospitals and evaluated on the fifth. Differences in precision were observed due to the different devices and different surgical techniques used. The recognition precision of the surgical phases varied from 79 to 90.6%.

Similarly, Cheng et al. conducted a multicenter study with the aim of developing an AI capable of recognizing the surgical phases of LC [48]. The authors used 90 videos (from which they extracted 156,584 frames) of LC to train the AI and another 60 (from which they extracted 15,395 frames) to evaluate it. The videos came from 4 Chinese hospitals. Both young and experienced surgeons performed LCs, to analyze the difference in surgical stages. LC was divided into six surgical phases: establishing access (EA); lysis of adhesion (AL); mobilize the hepatocystic triangle (MHT); dissect the gallbladder from the liver bed (DGB); clear the operational region (COR); extract the gallbladder (EG). The authors included cases of acute cholecystitis. In cases of acute cholecystitis, the surgical phases lasted longer. The authors used one AI to identify surgical phases and another AI to classify surgical phases from the perspective of temporal sequences. The AI achieved an accuracy of 91.05%.

### Limits of AI applied to LC

Most of the studies analyzed presented common limits.

Recruiting expert surgeons capable of annotating an AI system was the main challenge. In fact, to annotate frames from LC videos it is necessary to train the surgeons themselves in this task through specific training. The number of annotating surgeons for each study was often small (less than 10). This blurred the intersection regions within the expert-annotated heatmaps and potentially reduced the performance of the AI models. Furthermore, expert surgeons presented differences in their annotations during the AI training phase on the LC images proposed to them, caused by different personal experience.

Furthermore, in some cases, the AIs showed a variation in the accuracy of recognition of both safe and unsafe zones and surgical phases when given LC inputs derived from structures other than the one in which the AI had been trained. This could be caused both by the different surgical technique with which the different LCs are performed and by the different medical devices used during the LCs. Another explanation could be the small number of LC videos with which the AIs were trained (often only a hundred LPs). Furthermore, the classic rectangular shape of the ROI could affect the visual field of the operating surgeon during LC: in fact, some authors have reported a flickering in the dynamic images of the surgical field when the ROI was applied. In only one study analyzed, the authors replaced the rectangular ROI with tile-shaped safe and unsafe anatomic structure visualization technology (*YOLOv3*), capable of automatic repositioning and scaling to form anatomic landmarks. Furthermore, using this form of visualization technology, the authors observed a reduction in flickering of the anatomic structure of interest.

In addition, most studies were performed on LCs without complex situations such as acute and chronic cholecystitis, not allowing an adequate evaluation of AI in these contexts and in most studies, the dataset for training the AI came from a single center, preventing adequate generalization of the results.

Another limitation of the studies concerned the angle of the laparoscopic scope. In fact, the *GoNoGoNet system* identified the safe and unsafe areas only when Calot's triangle was framed by the laparoscopic optics together with the gallbladder and the liver. When vision magnification occurred during LC, the AI model misinterpreted the actual safe and unsafe zones. Furthermore, the presence of fumes and fluid loss (such as bile in case of gallbladder perforation) worsened the performance of AIs in terms of accuracy in recognizing safe and unsafe zones and surgical phases during LC. In other cases, AI models had difficulty recognizing structures of anatomic interest covered or hidden by adipose or fibrous tissue. The cause of this was both the absence of a sufficient number of annotations during the training phase of the AI by the annotating surgeons and the presence of anatomic boundaries which were consequently not well defined.

Another limitation common to most studies is that the evaluation of the technique for detecting CVS and of safety and danger zones during LC surgery using a heat map and comparing them with previous surgical images was limited to simulation using videos surgical, which had not reached operating room specifications. In only one study, the AI system was connected to the integrated endoscopic operating room (*EndoALPHA*; Olympus Corp.).

Finally, an investigation method for the prevention of BDI by AI has not currently been created, probably due to the still small number of studies conducted in this field of research. To conclude, AI can suggest safer dissection areas, but this does not imply that operating surgeons will actually incorporate such advice into their mental model, behaviors, and actions. This is because there are important design and implementation barriers to bringing AI into the operating room that have yet to be explored, such as usability, workflow, interface, data pipeline, and infrastructure. According to some authors, the majority of surgeons believed that AI would be beneficial for patient care but only a minority believed that it could improve decisions and intraoperative performance by requiring its on-demand use, with the possibility of deactivating it if necessary to reduce distractions due for example to ROI boxes. Although it is certainly advantageous to limit distractions in the surgical field, this functionality would probably reduce the usefulness of AI applied to LCs.

### Future perspectives

All the studies examined agree that AI has wide possibilities for improvement in future but that new studies are needed to confirm the advantages in the fields of application of this review. In many studies, AI has been helpful in recognizing the location of CD and EHBD, which may be helpful in avoiding BDI. Indiocyanin green (ICG) is currently used for this purpose during many LCs. However, the ICG has some disadvantages: the rare presence of patients with allergic reactions to its administration, the ICG-emission range wider than the real one, the limited emission time and the inability to show RS and S4. Unlike ICG, AI could present advantages: it has no impact on the patient and has no time limits in its intraoperative use. We can state that the simultaneous use of ICG and AI could potentially support the recognition of more accurate anatomic information during LC.

Another important application of these findings is in surgical training and performance evaluation and in the emerging field of automated coaching. Studies agree that the information detected by AI is objectively useful because it adds clinical meaning to the operating surgeon's assessment during LC. Therefore, in future, AI could be used both as a training model and as an intraoperative support for novice surgeons to recognize anatomic structures that are more difficult to interpret and thus reduce the risk of BDI and other complications of LCs. Indeed, several studies have evaluated the ability of AI models to recognize CSV in LC. This ability has been evaluated in several studies. Currently, AI models have been created that detect CVS with high accuracy in real time during LC. We can state that surgical safety could be improved by using image classification technology to identify the achievement of CVS during LC.

In future, it could improve the system for detecting anatomic structures of surgical interest. Currently, only in one study does the ROI have a shape that is not rectangular but rather dynamic tiles. A future perspective could be the implementation of the adoption of the ROI model with dynamic tiles to recognize anatomic structures that are difficult to recognize (such as when hidden by fat or fibrosis) or to reduce peripheral flicker of images during LC.

In only one of the studies examined, the AI system was integrated with the laparoscopic system of the operating room. This gave the operating surgeon the possibility of simultaneously observing the laparoscopic images on a monitor and the suggestions given by the AI on another monitor near the first. It is hoped that in future the number of AI models integrated into the laparoscopic systems of operating rooms can be increased, to allow the surgeon to simultaneously evaluate the indications of the AI and the laparoscopic images in real time.

## Conclusions

Our review suggests that during LC, AI can be used to identify safe and unsafe dissection zones and other anatomic structures. The possibility of contributing to the prevention of BDI and recognition of anatomic structures and surgical steps during LC was also evident, thanks to semantic and temporal segmentation. Furthermore, the AI provided important awareness to both novices and experts, prompting them to recognize anatomic landmarks related to BDI reduction. As more evidence emerges over time about the safety and effectiveness of using AI in the operating room, these automated computer vision tasks have the potential to increase performance and possibly be used in future for real-time decision support and other quality improvement initiatives.

However, large-scale evaluation is needed and the development of guidelines for both the annotation and clinical application of AI surgical support devices is also important.

## Data Availability

No additional data are available.

## Declarations

### Provenance

Not commissioned.

## References

[1] Hamet P, Tremblay J (2017) Artificial intelligence in medicine. Metabolism 69S:S36–S40. 10.1016/j.metabol.2017.01.011 10.1016/j.metabol.2017.01.011 28126242

[2] Howard J (2019) Artificial intelligence: implications for the future of work. Am J Ind Med 62:917–926 31436850 10.1002/ajim.23037

[3] Palomba G, Fernicola A, Della Corte M, Capuano M, De Palma GD, Aprea G (2024) Artificial intelligence in screening and diagnosis of surgical diseases: a narrative review. AIMS Public Health 11(2):557–576. 10.3934/publichealth.2024028 10.3934/publichealth.2024028 PMC11252578 39027395

[4] Akinrinmade AO, Adebile TM, Ezuma-Ebong C et al (2023) Artificial intelligence in healthcare: perception and reality. Cureus 15:e45594 37868407 10.7759/cureus.45594 PMC10587915

[5] Kaul V, Enslin S, Gross SA (2020) History of artificial intelligence in medicine. Gastrointest Endosc 92:807–812 32565184 10.1016/j.gie.2020.06.040

[6] Khalid MU, Laplante S, Masino C et al (2023) Use of artificial intelligence for decision-support to avoid high-risk behaviors during laparoscopic cholecystectomy. Surg Endosc 37:9467 37697115 10.1007/s00464-023-10403-4

[7] Keiler A, Pernegger C, Hornof R (1992) Laparoscopic cholecystectomy–current status. Wien Klin Wochenschr 104:29–38 1535168

[8] Fielding GA (1992) Laparoscopic cholecystectomy. Aust N Z J Surg 62:181–187 1532305 10.1111/j.1445-2197.1992.tb05459.x

[9] Shea JA, Healey MJ, Berlin JA et al (1996) Mortality and complications associated with laparoscopic cholecystectomy: a meta-analysis. Ann Surg 224:609–620 8916876 10.1097/00000658-199611000-00005 PMC1235438

[10] Strasberg SM (2002) Avoidance of biliary injury during laparoscopic cholecystectomy. J Hepatobiliary Pancreat Surg 9:543–547 12541037 10.1007/s005340200071

[11] Davidoff AM, Pappas TN, Murray EA et al (1992) Mechanisms of major biliary injury during laparoscopic cholecystectomy. Ann Surg 215:196–202 1531913 10.1097/00000658-199203000-00002 PMC1242421

[12] Soper NJ, Stockmann PT, Dunnegan DL et al (1992) Laparoscopic cholecystectomy the new ‘gold standard’? Arch Surg 127:917–921 1386505 10.1001/archsurg.1992.01420080051008

[13] Hugh TB (2002) New strategies to prevent laparoscopic bile duct injury—surgeons can learn from pilots. Surgery 132:826–835 12464867 10.1067/msy.2002.127681

[14] Way LW, Stewart L, Gantert W et al (2003) Causes and prevention of laparoscopic bile duct injuries: analysis of 252 cases from a human factors and cognitive psychology perspective. Ann Surg 237:460–469 12677139 10.1097/01.SLA.0000060680.92690.E9 PMC1514483

[15] Johnson-Mann CN, Loftus TJ, Bihorac A (2021) Equity and artificial intelligence in surgical care. JAMA Surg 156:509–510 33625504 10.1001/jamasurg.2020.7208 PMC8273554

[16] Loftus TJ, Upchurch GR, Bihorac A (2021) Building an artificial intelligence-competent surgical workforce. JAMA Surg 156:511–512 33688933 10.1001/jamasurg.2021.0045 PMC8273553

[17] Choi RY, Coyner AS, Kalpathy-Cramer J et al (2020) Introduction to machine learning, neural networks, and deep learning. Transl Vis Sci Technol 9:14 32704420 10.1167/tvst.9.2.14 PMC7347027

[18] Chen M, Decary M (2020) Artificial intelligence in healthcare: an essential guide for health leaders. Healthc Manag Forum 33:10–18 10.1177/0840470419873123 31550922

[19] Deo RC (2015) Machine learning in medicine. Circulation 132:1920–1930 26572668 10.1161/CIRCULATIONAHA.115.001593 PMC5831252

[20] Lo Vercio L, Amador K, Bannister JJ et al (2020) Supervised machine learning tools: a tutorial for clinicians. J Neural Eng 17:062001 10.1088/1741-2552/abbff2 33036008

[21] Ramesh AN, Kambhampati C, Monson JRT et al (2004) Artificial intelligence in medicine. Ann R Coll Surg Engl 86:334–338 15333167 10.1308/147870804290 PMC1964229

[22] Wiljer D, Hakim Z (2019) Developing an artificial intelligence-enabled health care practice: rewiring health care professions for better care. J Med Imaging Radiat Sci 50:S8–S14 31791914 10.1016/j.jmir.2019.09.010

[23] de’Angelis N, Catena F, Memeo R et al (2021) WSES guidelines for the detection and management of bile duct injury during cholecystectomy. World J Emerg Surg. 10.1186/s13017-021-00369-w 34112197 10.1186/s13017-021-00369-w PMC8190978

[24] Waage A, Nilsson M (2006) Iatrogenic bile duct injury: a population-based study of 152 776 cholecystectomies in the Swedish inpatient registry. Arch Surg 141:1207–1213 17178963 10.1001/archsurg.141.12.1207

[25] Strasberg SM, Hertl M, Soper NJ (1995) An analysis of the problem of biliary injury during laparoscopic cholecystectomy. J Am Coll Surg 180:101 8000648

[26] Manatakis DK, Antonopoulou MI, Tasis N et al (2023) Critical view of safety in laparoscopic cholecystectomy: a systematic review of current evidence and future perspectives. World J Surg 47:640–648 36474120 10.1007/s00268-022-06842-0

[27] Stefanidis D, Chintalapudi N, Anderson-Montoya B et al (2017) How often do surgeons obtain the critical view of safety during laparoscopic cholecystectomy? Surg Endosc 31:142–146 27142437 10.1007/s00464-016-4943-5

[28] Strasberg SM (2019) A three-step conceptual roadmap for avoiding bile duct injury in laparoscopic cholecystectomy: an invited perspective review. J Hepatobiliary Pancreat Sci 26:123–127 30828991 10.1002/jhbp.616

[29] Eikermann M, Siegel R, Broeders I et al (2012) Prevention and treatment of bile duct injuries during laparoscopic cholecystectomy: the clinical practice guidelines of the European Association for Endoscopic Surgery (EAES). Surg Endosc 26:3003–3039 23052493 10.1007/s00464-012-2511-1

[30] Pucher PH, Brunt LM, Davies N et al (2018) Outcome trends and safety measures after 30 years of laparoscopic cholecystectomy: a systematic review and pooled data analysis. Surg Endosc 32:2175–2183 29556977 10.1007/s00464-017-5974-2 PMC5897463

[31] Nijssen MAJ, Schreinemakers JMJ, Meyer Z et al (2015) Complications after laparoscopic cholecystectomy: a video evaluation study of whether the critical view of safety was reached. World J Surg 39:1798–1803 25711485 10.1007/s00268-015-2993-9

[32] Rawlings A, Hodgett SE, Matthews BD et al (2010) Single-incision laparoscopic cholecystectomy: initial experience with critical view of safety dissection and routine intraoperative cholangiography. J Am Coll Surg 211:1–7 20610242 10.1016/j.jamcollsurg.2010.02.038

[33] Sanford DE, Strasberg SM (2014) A simple effective method for generation of a permanent record of the Critical View of Safety during laparoscopic cholecystectomy by intraoperative ‘doublet’ photography. J Am Coll Surg 218:170–178 24440064 10.1016/j.jamcollsurg.2013.11.003

[34] Buddingh KT, Morks AN, Ten Cate Hoedemaker HO et al (2012) Documenting correct assessment of biliary anatomy during laparoscopic cholecystectomy. Surg Endosc 26:79–85 21792718 10.1007/s00464-011-1831-x PMC3242940

[35] Emous M, Westerterp M, Wind J et al (2010) Registering the critical view of safety: photo or video? Surg Endosc 24:2527–2530 20376491 10.1007/s00464-010-0997-y

[36] Kawamura M, Endo Y, Fujinaga A et al (2023) Development of an artificial intelligence system for real-time intraoperative assessment of the critical view of safety in laparoscopic cholecystectomy. Surg Endosc 37:8755–8763 37567981 10.1007/s00464-023-10328-y

[37] Mascagni P, Fiorillo C, Urade T et al (2020) Formalizing video documentation of the critical view of safety in laparoscopic cholecystectomy: a step towards artificial intelligence assistance to improve surgical safety. Surg Endosc 34:2709–2714 31583466 10.1007/s00464-019-07149-3

[38] Mascagni P, Vardazaryan A, Alapatt D et al (2022) Artificial intelligence for surgical safety: automatic assessment of the critical view of safety in laparoscopic cholecystectomy using deep learning. Ann Surg 275:955 33201104 10.1097/SLA.0000000000004351

[39] Madani A, Namazi B, Altieri MS et al (2022) Artificial intelligence for intraoperative guidance: using semantic segmentation to identify surgical anatomy during laparoscopic cholecystectomy. Ann Surg 276:363–369 33196488 10.1097/SLA.0000000000004594 PMC8186165

[40] Laplante S, Namazi B, Kiani P et al (2023) Validation of an artificial intelligence platform for the guidance of safe laparoscopic cholecystectomy. Surg Endosc 37:2260–2268 35918549 10.1007/s00464-022-09439-9

[41] Endo Y, Tokuyasu T, Mori Y et al (2023) Impact of AI system on recognition for anatomical landmarks related to reducing bile duct injury during laparoscopic cholecystectomy. Surg Endosc 37:5752–5759 37365396 10.1007/s00464-023-10224-5 PMC10322759

[42] Nakanuma H, Endo Y, Fujinaga A et al (2023) An intraoperative artificial intelligence system identifying anatomical landmarks for laparoscopic cholecystectomy: a prospective clinical feasibility trial (J-SUMMIT-C-01). Surg Endosc 37:1933–1942 36261644 10.1007/s00464-022-09678-w

[43] Fujinaga A, Endo Y, Etoh T et al (2023) Development of a cross-artificial intelligence system for identifying intraoperative anatomical landmarks and surgical phases during laparoscopic cholecystectomy. Surg Endosc 37:6118–6128 37142714 10.1007/s00464-023-10097-8

[44] Golany T, Aides A, Freedman D et al (2022) Artificial intelligence for phase recognition in complex laparoscopic cholecystectomy. Surg Endosc 36:9215–9223 35941306 10.1007/s00464-022-09405-5 PMC9652206

[45] Madni TD, Nakonezny PA, Barrios E et al (2019) Prospective validation of the Parkland Grading Scale for Cholecystitis. Am J Surg 217:90–97 30190078 10.1016/j.amjsurg.2018.08.005

[46] Madni TD, Leshikar DE, Minshall CT et al (2018) The Parkland grading scale for cholecystitis. Am J Surg 215:625–630 28619262 10.1016/j.amjsurg.2017.05.017

[47] Czempiel T, Paschali M, Keicher M, et al (2020) TeCNO: Surgical Phase Recognition with Multi-Stage Temporal Convolutional Networks. Lecture Notes in Computer Science (including subseries Lecture Notes in Artificial Intelligence and Lecture Notes in Bioinformatics) 12263 LNCS, pp 343–352

[48] Cheng K, You J, Wu S et al (2022) Artificial intelligence-based automated laparoscopic cholecystectomy surgical phase recognition and analysis. Surg Endosc 36:3160–3168 34231066 10.1007/s00464-021-08619-3

